# Empowering Patients in the Therapeutic Decision-Making Process: A Glance Into Behçet's Syndrome

**DOI:** 10.3389/fmed.2021.769870

**Published:** 2021-12-13

**Authors:** Diana Marinello, Federica Di Cianni, Alessandra Del Bianco, Irene Mattioli, Jurgen Sota, Luca Cantarini, Giacomo Emmi, Pietro Leccese, Giuseppe Lopalco, Marta Mosca, Angela Padula, Matteo Piga, Carlo Salvarani, Domenica Taruscio, Rosaria Talarico

**Affiliations:** ^1^Rheumatology Unit, Azienda Ospedaliero Universitaria Pisana, University of Pisa, Pisa, Italy; ^2^Associazione Italiana Sindrome e Malattia di Behçet, Pontedera, Italy; ^3^Department of Experimental and Clinical Medicine, University of Firenze, Firenze, Italy; ^4^Rheumatology Unit, Department of Medicine, Surgery, and Neurosciences, University of Siena, Siena, Italy; ^5^Rheumatology Department of Lucania, Rheumatology Institute of Lucania, San Carlo Hospital of Potenza and Madonna delle Grazie Hospital of Matera, Potenza and Matera, Italy; ^6^Rheumatology Unit, Department of Emergence Medicine and Transplantation (DETO), University of Bari Aldo Moro, Bari, Italy; ^7^Rheumatology Unit, Department of Medical Sciences and Public Health, AOU University Clinic and University of Cagliari, Cagliari, Italy; ^8^Rheumatology Unit, Azienda USL-IRCCS di Reggio Emilia and Università di Modena e Reggio Emilia, Modena, Italy; ^9^National Centre for Rare Diseases, Istituto Superiore di Sanità, Rome, Italy

**Keywords:** Behçet disease, patient empowerment, patient education, decision making process (DMP), rare disease (RD)

## Abstract

Behçet's syndrome (BS) represents a challenging condition, characterized by a variable spectrum of disease profile and associated with a significant limitation of the daily activities as well as a potential negative impact on relationships and psychological status. Considering also the complexity of the therapeutic management of BS, that often includes biological off-label treatments, the participation in the therapeutic decision-making process of the BS patients is essential to ensure the integration of the care process into the life of the patient. For this reason, the empowerment of BS patients represents a crucial need and the present work is aimed at fully exploring all the potential variables implicated in the BS patient empowerment, also highlighting major points to consider and concrete actions to be planned in the immediate future in order to implement a pragmatic facilitation of the patients' empowerment.

## Highlights

- The process of patients' empowerment needs to be addressed as a systematic approach and should ensure the involvement of multiple stakeholders in order to be really efficient and effective.
- Considering the rarity and complexity of BS, patients' empowerment can highly contribute to improve the lives of patients, caregivers and families living with the disease and it foresees to work for the optimization of patient-clinician communication, self-management, patient education, sharing of the therapeutic decision-making process, partnership in research and policy making.
- BS patients' organizations, BS healthcare professionals and policy-makers can play a crucial role in co-designing and co-creating new initiatives and projects aimed at joining forces and promoting patients' empowerment across the BS community.

## Introduction

According to the World Health Organization, patient empowerment is defined as “a process through which people gain greater control over decisions and actions affecting their health” (1) and for obvious reasons this process can be considered of certain efficacy only when approached at both individual and community level. Several elements have been reported so far as being fundamental to the patient empowerment and among them, patients' awareness of their role in the care process, the need for adequate knowledge enabling the engagement with the healthcare provider, the acquisition of specific skills and the existence of a facilitating environment definitely represent the mainstay of the empowerment process. Patients' empowerment means above all enablement, therefore patients and caregivers have to right to know, to be motivated, responsible and enabled to be part of the care process, also when the process involves research activities and medicine development (2–4).

Independently from its specific field of application, health policy makers should support and address this essential need, both if patient empowerment represents a goal to be reached and when it is adopted as a specific approach. At the same time, healthcare professionals have the duty to encourage and contribute to patients' empowerment also in clinical practice. This is particularly crucial in people living with rare diseases, in which knowledge related to diagnosis, treatment and complications is often limited and expertise is scattered (5, 6); however, in spite of the several challenges that rare diseases patients experience, the existing rare diseases patients' organizations facilitate the establishment or joining communities that play an essential role in providing the often-lacking information (7).

In the clinical environment of rare systemic autoimmune diseases, Behçet's disease (BS) represents a challenging condition, characterized by a variable spectrum of disease profile (8); while prevalent muco-cutaneous involvement and arthritis represent the main clinical features in patients with a benign disease subset, there are other patients who potentially develop sight or life-threatening manifestations, due to ocular, neurological or major vascular involvement (9). The relapsing nature of the disease can determine exacerbations and remission of symptoms over time and various demographic factors are considered predictable of poor outcome in the short and long-term, such as age at disease onset, duration of disease or gender. In fact, younger male BS patients are generally more suitable to have a more severe disease, due to an increased frequency both of morbidity and mortality, related to ocular, vascular and neurological involvement (8). Moreover, the chronic characteristics of the disease are strongly associated with a significant limitation of the daily activities as well as a potential negative impact on relationships with other people and psychological status (10, 11). Taking into account all these elements, it is clear that in order to manage in the most appropriate way the therapeutical approach according to disease activity, a very careful and tight control is strongly recommended in BS patients (12). Considering also the complexity of the therapeutic management of BS, that often includes biological off-label treatments, the participation in the therapeutic decision-making process of the BS patients is essential to ensure the integration of the care process into the life of the patient. For this reason, the empowerment of BS patients represents a crucial need to enable both this kind of integration and participation and to date, only few initiatives are ongoing to promote patients' empowerment in BS. Besides the fact that all patients living with BS could highly benefit in being included in the empowerment processes, it is also important to highlight that the different profiles of BS patients (such as age, type of organ involvement, severity of disease, etc.) may require targeted initiatives aimed at addressing the specific needs of the patient. Therefore, the present work is aimed at fully exploring all the potential variables implicated in the BS patient empowerment, also highlighting major points to consider and concrete actions to be planned in the immediate future in order to make real a pragmatic facilitation of the patients' empowerment.

## Domains to Address Patients' Empowerment in BS

### Sharing of the Therapeutic Decision-Making Process

Current evidence shows that adherence to therapy is usually higher in patients directly involved in the therapeutic decision-making process and, consequently, so is their outcome. In particular, lack of information concerning potential risks and benefits of the therapeutic options and inadequate communication between physicians and patients are some of the main risk factors for patients' discontinuation of treatment (13, 14).

Such observations revealed the need for new therapeutic decision-making processes, taking distances from the old paternalistic model which focused on simply informing patients about their treatment options rather than sharing them together to reach a common decision.

In facts the shared-decision making (SDM) process considers both the physician and the patient as its essential components: the former contributes with experience and the expertise on clinical guidelines and treatment's targets, while the latter expresses personal preferences and goals, as well as expectations from the treatment (15).

As reported in Table 1, the process comprises the following steps:

1. To describe the therapeutic options to patients and encourage them to actively participate into the conversation;
2. To explain the potential risks and benefits of each option;
3. To consider and evaluate patients' doubts and preferences;
4. To reach a shared decision on the best option;
5. To critically evaluate the decision and express any concern on the beginning of that specific medication (16).

**Table 1:** Points to consider to promote patients' empowerment in BS.

**Domains**	**Points to consider for patients' empowerment in BS**
Sharing of the therapeutic decision-making process	- Specific SDM models should be developed for BS. - Behçet's disease healthcare professionals should be trained in adopting shared decision-making models in BS.
The role of healthcare professionals in improving patients' empowerment in clinical practice	- Ensure the organization of training activities for healthcare professionals on how to communicate with patients and for patients to ensure an appropriate level of health literacy. - Co-create Patient Decision Aids dedicated to BS. -At hospital level, it is crucial to ensure a patient-centered approach during all phases of care dedicated to patients with BS (access to patient-clinician direct communication, to information on the disease, on the clinic, etc.).
Patient-clinician communication	- It is essential to promote training and education of healthcare professionals dealing with BS not only on the clinical aspects of BS, but also on how to communicate in general with the patient and on how to ensure a patient-centered and disease-specific communication related on BS. - Educational activities for patients should be focused on health literacy and on the specificities of BS. - Supporting the use of communication tools among BS clinics and patients' organizations including web-based ones, such as brochures, media platforms, workshops, open conferences could contribute to ensure an adequate access to information.
Self-management	- By joining efforts of the whole BS community of patients and healthcare professionals, it is desirable to identify a core set of areas of intervention in order to launch specific initiatives promoting BS self-management at local, national and international level.
The role of caregivers	- The empowerment of caregivers should also be ensured in order to improve the quality of life of both patients and caregivers. - While co-designing patient education programmes, dedicated initiatives should be specifically organized also for caregivers. - It is important to ensure access to information on BS also to caregivers.
Partnership in research	- In order to enable and encourage partnership among patients in the research process, support the creation of digital platforms dedicated to this aim. - Support the validation and standardization of co-designed outcome measures for BS research.
Patient education	- Specific patients' education programmes need to be developed for BS. - Any educational programme should be developed in co-design with BS patients' and BS patients' representatives in order to address their educational needs and priorities. - Caregivers and family members should also receive specific training and should participate in the co-design process.
Patients' empowerment and policy maker	- Promoting the creation of patients' organizations and federations dedicated to BS can support the empowerment of patients at different level and ensure the active participation of BS patients' representatives in policy making and other relevant initiatives.

The benefits gained by this approach include the beginning of a tailored therapy which the patient can actually benefit from the reduction of unwarranted health practice variations and, most importantly, the acknowledgment of the patients' right to participate in decisions involving their health. In addition, principles and strategies of SDM process in the therapeutic decisions may contribute to improve patients' adherence, as highlighted by the International Patient Decision Aid Standards Collaboration (17)^1^. However, currently, models of SDM tailored to BS are still lacking, probably also because the application of therapeutic SDM process in BS appears even more challenging as it implies different therapeutical approach, both traditional and new molecules which bring different clinical outcome according to the main disease manifestations (e.g., anti-IL-1 against muco-cutaneous disease, anti-IL-6 against neurologic disease etc.).

#### Points to Consider

- Specific SDM models should be developed for BS.
- BS healthcare professionals should be trained in adopting SDM models in BS.

### The Role of the Healthcare Professionals in Improving Patients' Empowerment in Clinical Practice

The processes of patient empowerment in clinical practice are strictly related to the concepts of patient-centered care and participation in the decision-making process (18). These two concepts can be achieved also with the contribution of the healthcare professionals by ensuring an appropriate efficient communication and open dialogue among patients and healthcare professionals and encouraging an active participation of patients in their care. Healthcare professionals can in fact play a crucial role in promoting the empowerment of patients, not only by providing detailed information on the disease and on the treatment options available, but also involving patients in the decisions that affect their quality of life. Establishing a robust relationship among healthcare professionals and patients and involving patients in a shared decision process are also essential to promote patient's empowerment and have proved to contribute to a better clinical outcome (19).

One of the tools that are currently available to support patients in actively participating to the decision-making process is the Patient Decision Aids (PDA) (20, 21), that are defined as “tools designed to help people participate in decision making about health care options. They provide information on the options and help patients clarify and communicate the personal value they associate with different features of the options.” Thanks to the development of PDA, patients and healthcare professionals can actively share their point of view regarding the best treatment options and thus representing a tangible and powerful tool of patients' empowerment. Even if some PDA are available for some rheumatic diseases (22), so far, no PDA are available in BS and in order to promote and support the participation of patients in decision-making process, co-creating PDA specific for BS should be a priority of the BS community.

Another important aspect related to the role of the healthcare professionals in improving patients' empowerment in clinical practice, is the organization of specific initiatives at hospital level. These include ensuring access to specific information on BS and on the BS clinic to patients (for example on the website of the hospital, providing printed leaflets at the clinic, etc.) and organizing on-line communication channels among patients and healthcare professionals (such as dedicated email addresses, FAQs, etc.), as well as ensuring patients' access to their medical records. In addition, the provision of telemedicine services for rare diseases such as BS is also particularly important and can highly contribute to empowering patients, especially considering the scattered knowledge existing worldwide.

It is important to highlight that the healthcare professionals that can contribute to patients' empowerment are not only clinicians, but also nurses, occupational therapists, physiotherapists, social workers, psychologists, etc. For this reason, specific training on communication and on how to ensure the active participation of patients in the shared-decision making process should be dedicated to all these different professionals, that can highly contribute to the empowerment of patients.

#### Points to Consider

- Ensure the organization of training activities for healthcare professionals on how to communicate with patients and for patients to guarantee an appropriate level of health literacy.
- Co-create Patient Decision Aids dedicated to BS.
- At hospital level, it is crucial to ensure a patient-centered approach during all phases of care dedicated to BS patients (access to patient-clinician direct communication, to information on the disease, on the clinic, etc).

### Patient-Clinician Communication

Communication between the healthcare professional and the patient is a complex and interactive process. Thanks to an interactive communication, doctors and patients can share precious details regarding a medical history, co-identify signs and symptoms necessary for a correct diagnosis and share decisions on treatments based on the assessment of the risk/benefit of the therapy.

The main factors correlated to an appropriate patient-clinician communication include patient participation, efficient SDM, treatment satisfaction and building a mutual trust relationship (23). Evidence from the literature seems to suggest that a good patient-clinician communication means better global health, less organ damage, lower disease activity and fewer medication side effects (24–29). Other works focused on communication in chronic diseases, assessing that doctor's beliefs, attitudes and style impact on the relationship built with the patient and, as a result, doctors who are more informative, show more sensitivity to patients' concerns and offer more reassurance and support, tend to have patients satisfied with care and committed to treatment recommendations (30, 31). Moreover, the use of a simple, understandable and non-technical language appears essential for an efficient patient-clinician communication, as well as non-verbal communication seems to be predictive for patients' satisfaction: in fact, patients' poor understanding, in some cases due to the use of medical terminology during consultations, can cause anxiety, fear and disappointment (32).

Communication between patients and their doctors has been greatly revolutionized by the narrative medicine, a branch of health humanities that employs narratives of patients in clinical practice, research and education as a way to promote healing process (33, 34). In addition, it is of great interest the narrative reciprocity: the narrative and potentially reciprocal nature of attention in health care. “Narrative reciprocity might enable not only so-called shared decision-making and patient autonomy. It might open the door to mutual acknowledgment of the value of each participant's beliefs and habits” (35).

Moreover, the widespread use of web-sites, forums and social networks provides patients with direct access to the medical and scientific information available online. Patients use these channels as a tool of personal participation, even directly interacting with health structures and professionals by digital platforms. In this scenario where patients increasingly pursue to play an active role in their care process, patient-reported outcomes (PROs) represent a remarkable tool for clinicians to learn and understand patients' experiences and needs. Indeed, PROs quantify health outcomes reported directly by patients, without external interpretation or inputs (36, 37). A recent study evaluated the use of PROs in rheumatoid arthritis consultations and showed that PROs were feasible, increased a shared understanding of how disease affects patients' function in daily life, encouraged communication and shared decision-making and eventually resulted in high patient satisfaction and treatment confidence. Moreover, PROs helped clinicians to identify new symptoms and adjust treatment as needed (38). Therefore, an effective patient-clinician communication potentially improves three aspects of empowerment:

1. Level of knowledge of disease and its implications;
2. Ability to control and monitor treatment progress, treatment adherence and disease-related lifestyle adjustments;
3. Active participation in interviews and better level of preparation for consultations with HCPs.

#### Points to Consider

- It is essential to promote training and education of healthcare professionals dealing with BS not only on the clinical aspects of BS, but also on how to communicate in general with the patient and on how to ensure a patient-centered and disease-specific communication related on BS.
- Educational activities for patients should be focused on health literacy and on the specificities of BS.
- Supporting the use of communication tools among BS clinics and patients' organizations including web-based ones, such as brochures, media platforms, workshops and open conferences could contribute to ensure an adequate access to information.

### Self-Management

The concept of self-management in the process of empowering patients arises from two main unmet needs: the health care system's difficulty in sustaining the efforts and costs of dealing with chronic conditions and rare diseases, and patients' need to develop a higher self-awareness of their condition.

A successful process of self-management is composed by different coexisting factors:

- Predisposing factors include motivation, self-efficacy and self-confidence showed by patients playing an active role in their health decision-making process;
- Reinforcing factors comprise family, patient organizations and health care professionals;
- Enabling factors are mainly represented by problem solving skills and access to healthcare information, both digital and not digital (39–41).

Self-management can be defined as the knowledge and skills that patients can acquire to better live with their condition, including the confidence in dealing with treatments management (e.g., for BS, suspension of immunosuppressive therapy in case of infection) and the be able to identify symptoms or signs needing immediate medical attention, emotional management, etc. In addition, health care professionals could strongly support the self-management by providing education and adequate information related to the disease and its management, in order to practically increase patients' skills in co-managing their health issues (42).

The recently published EULAR recommendations for the implementation of self-management strategies in inflammatory arthritis also offered a definition of self-management, that is the ability of the individual to deal with symptoms, treatment, lifestyle changes and psychosocial and cultural consequences of their condition. According to this work, self-management is inspired by two key themes: to achieve independence of the patient, the former, and the idea that self-management should be supported by others (e.g., family, patients organizations, healthcare professionals), the latter. Therefore, it becomes clear how EULAR found an integration between self-management and self-management support (39).

Other skills that patients should learn and improve for an efficient self-management approach were also previously identified (39, 40); some examples are offered by patient education, active involvement in problem solving/goal setting/decision-making, active interaction with significant others (healthcare professionals, family, patients' organizations), medication management, enhancing resource utilization (community, digital healthcare etc.), improving stress management (also cognitive behavior therapy, if needed), healthy behaviors (e.g., regular physical activity, diet, body weight control, quitting smoking) and managing work duties.

On the contrary, little evidence is currently available on the potential self-management strategies in BS patients. An important starting point is the identification of the factors mostly affecting people suffering from BS. In these regards, three works described BS patients' specific needs dividing them into four main domains (43, 44):

1. Sign and symptoms: mucocutaneous manifestations (especially oral and genital ulcers), pain, vision issues, fatigue and sleep disturbances;
2. Functioning: impact of the disease on speech and vision, lack of energy for daily activities, adaptation skills and self-care;
3. Psychological profile: impact on emotions and emotional management techniques;
4. Social impact: ability to socialize, impact on familial relationships.

Taking into account the evidence already available, it appears clear that more attention should be dedicated to the self-management of BS and to do that, it would be crucial to identify, together with BS patients, a core set of areas of intervention that can concretely stimulate the cultural change needed to join efforts and work at multi-stakeholder level on this goal.

#### Points to Consider

- By joining efforts of the whole BS community of patients and healthcare professionals, it is desirable to identify a core set of areas of intervention in order to launch specific initiatives promoting BS self-management at local, national and international level.

### The Role of Caregivers

Caregivers can be defined as “a person who gives care to people who need help taking care of themselves” (45), thus including parents, partners, friends, members of the family or healthcare professionals. To date, little attention has been given to the role that caregivers play in rare diseases and even less attention to their empowerment. Besides the launch of a survey dedicated to BS caregivers, very few data are available on the role played by BS caregivers in empowering patients and on the actual need of devoting more efforts toward the empowerment of BS caregivers.

Caregivers have an essential role in the life of BS patients, as they support the care of patients in the wider and most complex aspects, ranging from the management of treatments on a daily basis to supporting the wellbeing of patients. In its systemic and rare nature, BS caregivers can range from parents of a child affected by BS to partners, friends or family members of adult/elderly BS patients. Despite the importance of the role of caregivers, the burden of BS caregivers is still not fully explored and BS caregivers are not always appropriately informed and supported.

Therefore, it is important to mention that one of the most urgent needs in BS is the organization of educational activities dedicated to BS caregivers, in order to empower them in providing appropriate care to their patient, in knowing better the disease and the treatment options available, as well as in taking care of their own quality of life (46).

The introduction of healthcare professionals that can also support patient and caregiver information and education, such as specialized nurses, can also highly contribute to empowering caregivers.

In clinical practice, the point of view of caregivers should be also considered, especially in terms of quality of life and of disease burden and dedicated information should be made available also to BS caregivers (information on BS, on how to access the clinic, patient/caregiver-clinician communication channel, etc.).

#### Points to Consider

- The empowerment of BS caregivers should also be ensured in order to improve the quality of life of both patients and caregivers.
- While co-designing patient education programmes, dedicated initiatives should be specifically organized also for BS caregivers.
- It is important to ensure access to information on BS also to caregivers.

### Partnership in Research

Health research landscape is increasingly changing. Researchers historically considered themselves as “gatekeepers” as they decide the objectives of their works and how to measure the reliability of their data; moreover, usually the results are shown and discussed on scientific papers and during conferences exclusively dedicated to healthcare professionals. Therefore, without patients' involvement in research, the paradigm researcher-gatekeeper fails to capture what is really important from the patients' point of view and at catching many key points like the evaluation of health outcomes from the patient's point of view.

In this regard, two conditions should be met for research to qualify as patient-oriented:

1. Patients are involved as research partners with multidisciplinary and transdisciplinary team members along a continuum in addressing patient priorities and/or planning research (e.g., data collection and analysis, interpretations, diffusion, dissemination and application of results).
2. Studies aim to (i) address outcomes deemed important by patients; (ii) have a direct impact on at least 1 of the following targets: patient health and experience, health professionals' practice, health care services and policies; or (iii) achieve both (i) and (ii) (47, 48).

As far as BS is concerned, a systematic review assessed that outcome measures used so far still need to be validated and standardized (49), suggesting they might benefit from an active partnership between researchers and patients in the research process. Although some steps forward have been covered by promoting patients' involvement in core set outcome definitions and outcome measurements development (50, 51), there is still a long way to go.

#### Points to Consider

- In order to enable and encourage partnership among patients in the research process, support the creation of digital platforms dedicated to this aim.
- Support the validation and standardization of co-design co-designed outcome measures for BS research.

### Patient Education

Patients' education can be considered as one of main principles behind patients' empowerment, both in terms of knowledge and awareness on the disease and in terms of rights and responsibility that the patient has in the care process (52).

Besides the many initiatives that are already ongoing at European and National level (53), the high need of patient education for rare diseases is continuously highlighted in many different contexts. With regards to BS, the initiatives currently in place to support patient education are very limited. Among those, BehçeTalk (54) was recently launched in Italy by the Behçet Clinic of Pisa and National Association of Behçet disease and Behçet-like-Odv (Simba) as a patient education programme for patients, families and caregivers living with BS. The programme is offering educational webinars on the different aspects of the disease, as well a parallel programme that foresees support groups coordinated by a psychologist with specific expertise in BS. Ever since the very beginning of the programme, patients and clinicians dealing with BS have expressed the need to extend this programme also outside the Italian borders and for this reason, the programme will soon be launched worldwide also in English and in other languages. This experience has demonstrated that the BS patient community has identified an emerging unmet need in patient's education.

Considering the specificities of BS, it is easy to imagine how important it is to provide a specific education on the disease to BS patients and how this will inevitably lead to empower them not only in participating in the decision-making processes related to their care, but also in the self-management and in improving their daily life. Knowing the therapeutic options available, understanding how to be adherent to treatment, learning how to manage the disease in the daily life are in fact, concepts that could provide a considerable added value in the life of the patients, especially since many treatments adopted in BS are prescribed off-label and require specific adherence protocol that the patient needs to know in order to ensure their efficacy. An important principle needed in patients' education, is related to ensuring that any educational programme or activity is developed in co-design with patients and patients' representatives. As confirmed in the BehçeTalk programme, involving patients and patients' representatives in the development of patient education programmes ensures that the needs and the priorities of patients are being addressed and the educational activity are tailored to what patients consider important.

Parallelly, the need for education is focused not only on training patients on their disease, but also, as highlighted in other disease areas (55), on providing similar knowledge also to the ones that live and support the patients during their journeys: their family caregivers. The burden of the disease and quality of life are often not properly addressed in BS caregivers and also for this reason, they should be included in the design of educational programmes and specific educational activities should be also addressed to them.

#### Points to Consider

- Specific patients' education programmes need to be developed for BS.
- Any educational programme should be developed in co-design with BS patients' and BS patients' representatives in order to address their educational needs and priorities.
- Caregivers and family members should also receive specific training and should participate in the co-design process.

### Patient's Empowerment and Policy Maker

The process of patients' empowerment is part of a holistic approach that involves patients and caregivers/healthcare professionals at both individual and community level (56). Patients' empowerment has, in fact, two different but complementary dimensions: individual empowerment and collective empowerment. Collective patients' empowerment enables a community to express their needs and most of all, can facilitate the involvement of patients' representatives in policy-making aimed at shaping healthcare systems while addressing those needs. Ensuring patients' empowerment and involvement at policy-making level can in fact support the co-design of healthcare systems and services that are more patient-centered and that can be more effective and efficient.

The inclusion of patients in policy making and in the co-design of care services is usually ensured thanks to the representation of patients from patients' organizations (POs). POs can represent a community expressing their needs and priorities and act as stakeholder in the co-design of health policies, while advocating at political and social level to address those needs. With regards to BS, some BS POs are currently active at different levels [such as Simba Odv in Italy (57), Behçet UK (58), ABSA in America (59), etc.] and are often participating in important initiatives. In addition, through federations of rare disease POs, such as EURORDIS (60), BS POs are also (directly or indirectly) involved in policy making. However, many geographical areas are still lacking a dedicated PO for BS, and it is desirable, that more national PO and federations can be founded around the world in order to build a stronger community of patients, families, healthcare professionals and policy-makers that can join forces and co-create new knowledge and new policies to better address the needs of the BS community.

#### Points to Consider

- Promoting the creation of patients' organizations and federations dedicated to BS can support the empowerment of patients at different level and ensure the active participation of BS patients' representatives in policy making and other relevant initiatives.

## Case Scenario

Rebecca is 35 years old and she received a diagnosis of BS when she was 19 years old. After the diagnosis, she searched “Behçet's syndrome” on the internet and she felt overwhelmed by all the information found. At this stage, she found out about the national patients' organization dealing with BS and she had the opportunity of discussing her diagnosis, her symptoms, her daily life with other BS patients. Thanks to the patients' organization, she was referred to an expert center in her country. The healthcare professionals in the center provided her with informative material on the disease and on the treatment she was prescribed. However, many issues were still not completing clear to her, such as how to self-manage the treatment and when to suspend it, which were the possible side effects, etc. After discussing her concerns with the patients' organization, Rebecca was encouraged to discuss this further with her clinician and to participate in an educational program dedicated to her disease. Rebecca attended different sessions of the educational program on how to self-manage her treatments; thanks to her efforts, to the educational program and to the role played by the patients' organization, Rebecca felt more empowered and addressed her concerns during the next consultation, discussing how to self-manage her treatment. The joined efforts of the different stakeholders involved in the empowerment of Rebecca, have contributed to improving her knowledge of the disease and the treatment, as well as in enabling her in being actively involved in her therapeutic decision-making processes.

## Conclusions

The process of patients' empowerment needs to be addressed as a systematic approach and should ensure the involvement of multiple stakeholders in order to be really efficient and effective. Considering the rarity and complexity of BS, patients' empowerment can highly contribute to improve the lives of patients, caregivers and families living with the disease. Specific domains to be addressed in order to promote patients' empowerment in BS include patient-clinician communication, self-management, patient education, sharing of the therapeutic decision-making process, partnership in research and policy making, in which not only the individual patients, but also healthcare professionals and caregivers can strongly contribute.

In this scenario, BS patients' organizations, BS healthcare professionals and policy-makers can play a crucial role in co-designing and co-creating new initiatives and projects aimed at promoting patients' empowerment across the BS community. Joining forces across the whole community is, in fact a condition sine qua non for the implementation of a cultural change toward a new multi-dimensional and multi-stakeholder approach in the management of BS.

## Data Availability Statement

The original contributions presented in the study are included in the article/supplementary material, further inquiries can be directed to the corresponding author.

## Author Contributions

RT, DM, and MM conceived the paper. RT, DM, and FDC wrote the manuscript. All authors were involved in the discussion and in the agreement procedure that provided the points to consider. All authors repeatedly edited the manuscript and approved the final version.

## Conflict of Interest

The authors declare that the research was conducted in the absence of any commercial or financial relationships that could be construed as a potential conflict of interest.

## Publisher's Note

All claims expressed in this article are solely those of the authors and do not necessarily represent those of their affiliated organizations, or those of the publisher, the editors and the reviewers. Any product that may be evaluated in this article, or claim that may be made by its manufacturer, is not guaranteed or endorsed by the publisher.

## Footnotes

^1^ http://ipdas.ohri.ca/index.html
